# Comparative effects of different loads of aerobic exercise on lipid metabolism in MASLD rats: a perspective from the gut-liver axis

**DOI:** 10.3389/fmed.2025.1609751

**Published:** 2025-07-15

**Authors:** Deng Dongkun, Jiang Qingfeng, Li Chang, Lin Yunhua, Shi Jiaming, Liu Yufei, Xu Lin

**Affiliations:** College of Sports and Human Sciences, Graduate School, Harbin Sport University, Harbin, China

**Keywords:** MASLD, exercise training, lipid metabolism, bile acid pathway, gut-liver axis

## Abstract

**Objective:**

Exercise training has been shown to be effective in ameliorating obesity-related diseases, but the therapeutic effects of different loads of exercise on metabolic dysfunction-associated steatotic liver disease (MASLD) as well as the underlying mechanisms by which exercise is based on the enterohepatic axis and thus alleviates MASLD are still unclear. Therefore, the present study aimed to clarify the optimal exercise load for improving MASLD and to reveal its molecular mechanisms in the treatment of metabolic-associated fatty liver disease (MASLD) in the context of the enterohepatic axis.

**Methods:**

Forty male rats were randomly divided into two groups: NFD (*n* = 8) and HFD (*n* = 32). The rats in the NFD group were fed a normal chow, while those in the HFD group were fed a high-fat chow. Following an eight-week period of observation, the rats in the high-fat diet (HFD) group were separated into four further groups for the purpose of analysis: (1) LEH (low-load aerobic exercise)-8; (2) MEH (medium-load aerobic exercise)-8; (3) HEH (high-load aerobic exercise)-8; and (4) HFD-8. At the conclusion of the experiment, blood, liver, and ileum samples were collected for analysis of the rats’ baseline conditions, hepatic lipid metabolism, bile acid pathway and gut microbiota, and synthesis of analyses.

**Results:**

The development of lipid metabolism disorders, insulin resistance, and hepatic steatosis in MASLD rats was improved to different degrees in all three exercise modes. It also restored the high-fat diet (HFD)-induced intestinal barrier dysfunction and balanced the homeostasis of the gut-liver axis. Aerobic exercise also upregulated bile acid-related gene expression modulated butyrate-producing bacterial taxa, and adjusted the abundance of butyrate-generating bacteria.

**Conclusion:**

Compared with low-load aerobic exercise, medium- and high-load aerobic exercise was more beneficial in modulating lipid metabolism dysfunction in MASLD rats, and to some extent, high-load aerobic exercise was superior to medium-load aerobic exercise.

## Graphical abstract

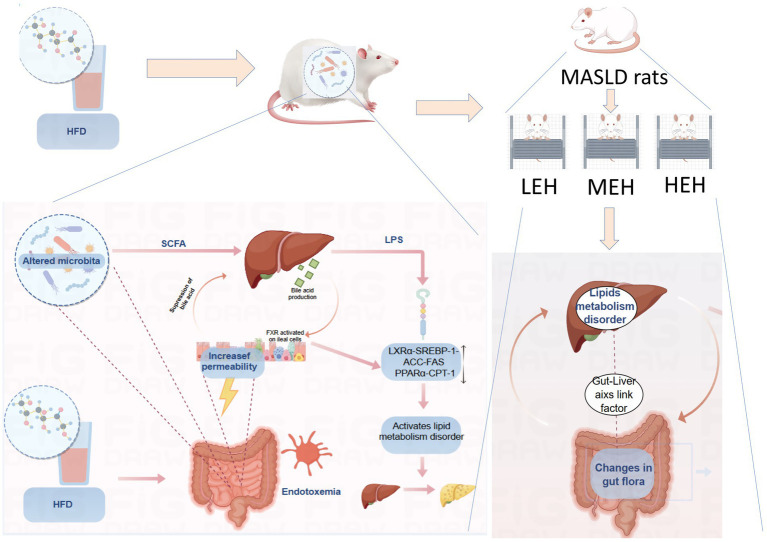

## 1 Introduction

Abnormal lipid metabolism is a clinical feature of most patients with metabolic abnormality-associated steatohepatopathy (MASLD), and the metabolic processes induced by a high-fat diet (HFD) can cause oxidative stress in the mitochondria and endoplasmic reticulum as well as inducing *de novo* lipogenesis and inflammation, which leads to disorders of lipid metabolism, a process that accelerates the development of MASLD (1, 2). Lipid metabolism is regulated by a variety of nuclear factors and genes (3), among which hepatic X receptor *α* (LXRα) is a class of nuclear receptors that regulate cholesterol metabolism and efflux, and can regulate cholesterol, bile acid and lipid metabolism during normal physiological processes. Studies have shown that, as a regulator of hepatic lipid regeneration, LXRα is involved in the pathogenesis of hepatic steatosis in patients with MASLD (4), and when LXRα overexpression activates the important regulator of adipogenesis, sterol regulatory element binding protein 1c (SREBP-1c), and fatty acid synthase, which controls fatty acid synthesis (fatty acid synthase, FAS), which controls fatty acid synthesis, a large amount of fatty acid streams pool in the liver, thereby inducing MASLD (5). Meanwhile, several studies have found a significant negative correlation between serum high-medium density lipoprotein cholesterol (HDL-C) levels and hepatic steatosis in MASLD patients (6). It has been demonstrated that HDL-C has potent anti-inflammatory properties that can alleviate lipopolysaccharide (LPS)-induced inflammation *in vitro* and *in vivo*, thereby delaying the malignancy of MASLD (7).

On the other hand, it has been extensively demonstrated that the gut microbiota increases host energy production from digested food, alters choline metabolism, and regulates the enterohepatic circulation of bile acids (BAs) (8, 9). Disturbances in the gut microbiota can lead to a reduction in the synthesis of secondary bile acids and impact gut barrier function. Increased intestinal permeability has been found in mice on high-fat or choline-deficient diets and in patients with MASLD (10), and microbial disorders and enhanced intestinal permeability expose the liver to gut-derived bacterial metabolites, leading to chronic endotoxemia and associated changes in the gut-hepatic axis. Migration of gut bacteria and their products to the liver via the gut-liver axis leads to disorders of lipid metabolism and elicits a range of immune system and inflammatory responses, which in turn lead to the development and formation of MASLD (11). Therefore, the protection of gut microecology informs the classical prediction of MASLD and suggests new therapeutic targets.

Currently, there is no standard treatment for MASLD, and some studies have demonstrated that lifestyle improvements have a significant ameliorative effect on MASLD (12). The American Association for the Study of Liver Diseases (AASLD) and a number of studies have also demonstrated the many efficacies of exercise for MASLD (13, 14). It has been found that long-term appropriate exercise can regulate lipid composition, reduce serum total cholesterol (TG), low-density lipoprotein (LDL-C), increase the level of HDL-C, improve lipid metabolism, and has a comprehensive and all-around character in terms of therapeutic effect (15, 16). Meanwhile, aerobic exercise can increase the levels of P-AMPK and p-AMPK/AMPK in liver tissues of rats on high-fat diet, and activated adenosine monophosphate-activated protein kinase phosphorylation (AMPK) can effectively inhibit the key enzymes of lipid synthesis as well as the expression of SREBP-lc and FAS, which can effectively decrease fat synthesis and inhibit the accumulation of visceral fat, thus preventing and controlling MASLD (17, 18). Zhang Huijie et al. randomly assigned participants to moderate-intensity exercise groups and to moderate exercise or no exercise, and found that vigorous and moderate exercise were equally effective in lowering intrahepatic triglyceride levels, and both reduced body weight (19), similar to previous studies (20). However, Ruan Ling’s study on NAFLD rats found that there was no significant difference in body weight among the exercise groups. However, moderate-intensity exercise was more effective in improving antioxidant capacity and inhibiting hepatocyte apoptosis (21). Therefore, there is no consensus on the most effective modality or intensity for treating MASLD (22).

In contrast to the findings of previous studies, the present study elucidated the intestinal and hepatic crosstalk mechanisms of different loading exercises in promoting MASLD lipid metabolism from the perspective of intestinal and hepatic axes. Furthermore, this study compared three distinct loading exercises. This approach may offer a scientific and health-conscious alternative to conventional exercise for the management of MASLD. Consequently, further elucidation of the most efficacious exercise modalities is both scientifically and practically advantageous. Such elucidation should reveal the molecular mechanisms by which exercise regulates metabolic syndrome-associated liver disease (MASLD) on the basis of the gut-liver axis. Furthermore, identification and exploration of the key gut microbes that contribute to the prevention and treatment of MASLD is imperative.

## 2 Methods

### 2.1 Experimental animals, chemicals and antibodies

Forty 6-week-old SPF-grade healthy male SD rats (180 ± 20 g) were selected for this study, provided by Liaoning Changsheng Biotechnology Co. Ltd., with Laboratory Animal Production License No. [SCXK (Liao) 2020–0001] and Use License No. [SYXK (Hei) 2018–007]. The experimental procedures were housed in standard Special Pathogen Free (SPF) conditions in separate cages at the Animal Breeding Center of Formulary of Heilongjiang University of Traditional Chinese Medicine. All experimental animals were placed in the same environment with room temperature maintained at 24 ± 2°C, relative humidity of about 45–65%, 12 h/12 h circulating lighting day and night, and free access to food and water. All animal experiments in this study were approved by the Academic Ethics Committee of Harbin Institute of Physical Education and Sports, with the ethical approval number IACUC (2024038).

The following chemicals were obtained from Servicebio (Wuhan, China): phenylmethanesulfonyl fluoride (PMSF), radioimmunoprecipitation assay (RIPA) lysis buffer, enhanced chemiluminescence (ECL) reagent, polyvinylidene fluoride (PVDF) membranes, and an SDS-PAGE gel preparation kit. Rabbit monoclonal primary antibodies against: phosphorylated AMP-activated protein kinase (pAMPK); liver X receptor *α* (LXRα); sterol regulatory element binding protein 1c (SREBP-1c); carnitine palmitoyltransferase 1 (CPT-1); peroxisome proliferator-activated receptor *α* (PPARα); cytochrome P450 2E1 (CYP2E1) were purchased from Servicebio. Monoclonal antibodies targeting *β*-actin and Lamin B were also sourced from Servicebio. Fatty acid synthase (FAS) was obtained from Abcam (Cambridge, MA, USA), and acetyl-CoA carboxylase (ACC) was acquired from Proteintech Group, Inc. (Wuhan, China). Horseradish peroxidase (HRP)-Servicebio provided conjugated goat anti-rabbit and mouse immunoglobulin G (IgG) secondary antibodies. Antibodies targeting intestinal barrier-related proteins were purchased from Wuhan Three Eagles Biotechnology Co., Ltd. (Hubei, China). Enzyme-linked immunosorbent assay (ELISA) kits for LPS, interleukin-1β (IL-1β), interleukin-6 (IL-6), tumor necrosis factor-*α* (TNF-α), and leptin were obtained from Mlbio (Shanghai, China).

### 2.2 Interventions

After 1 week of acclimatization, 40 SD rats were randomly divided into 5 groups of 8 rats each, and fed different experimental feeds: the control group (*n* = 8) was fed a normal fat feed with a fat content to energy ratio of 10 kcal% purchased from Liaoning Changsheng Biotech Co., Ltd. and the experimental feed production license No.: SCXK (Liao) 2015–003, which is the national standard rodent dry. The feed was a national standard dry rodent feed; high-fat control rats and rats in the exercise group (*n* = 32) were fed a high-fat feed with a fat content of 45 kcal% (China, Beijing, Huafukang Bioscience Co., Ltd., H10045). The steatosis disorder was stimulated for 8 consecutive weeks. The success of modeling was determined according to the blood biochemical indexes and liver histology of the rats. After successful modeling, the rats in the high-fat diet group were randomly divided into the HFD group, low-load aerobic exercise group (LEH), medium-load aerobic exercise group (MEH), and high-load aerobic exercise group (HEH), and the rats in the exercise group were subjected to the exercise interventions respectively: using the ZS-PT-III (Zongshi Dichuang Science and Technology Development Limited Liability Company, Beijing, China) running table, the rats were subjected to three kinds of running table exercise for 8 weeks, respectively, with different loads of exercise. During the 8-week experimental period, all rats were weighed weekly, and feed intake was recorded, and the training program model (23, 24) was slightly adjusted with reference to the method of Ortega-Santos (25) and others, as shown in Table 1. Finally, rats were fasted overnight and executed by injection of ether, blood was taken from the abdominal aorta, serum was extracted by centrifugation, and tissues such as liver, ileum, subcutaneous fat, and epididymal fat were taken and weighed. Liver tissues were immediately transferred to a formaldehyde solution to assess morphological changes, then frozen in liquid nitrogen and stored at - 80°C for the following assays.

**Table 1:** Exercise training program for rats.

LEH			
Experimental period	Time	Speed (m/min)	Training time (min)
Week 1	Day 1	15	5
	Day 2	16	10
	Day 3	17	15
	Day 4	18	20
	Day 5	19	25
	Day 6	20	30
2–8 weeks	1–6 Day	20	30

MEH			
Experimental period	Time	Speed (m/min)	Training time (min)
Week 1	Day 1	15	30
	Day 2	16	40
	Day 3	17	45
	Day 4	18	50
	Day 5	19	55
	Day 6	20	60
2–8 weeks	1–6 Day	20	60

MEH			
Experimental period	Time	Speed (m/min)	Training time (min)
Week 1	Day 1	15	60
	Day 2	16	70
	Day 3	17	75
	Day 4	18	80
	Day 5	19	85
	Day 6	20	90
2–8 weeks	1–6 Day	20	90

### 2.3 Glucose and insulin tolerance analyses

GTT and ITT were performed at the end of the exercise period (weeks 15–16) to evaluate sustained metabolic improvements, with tissue collection following the tests to correlate functional outcomes with histological/biochemical changes. At weeks 15 and 16 of the trial, the rats were intraperitoneally administered glucose (2 g/kg) and insulin (0.75 U/kg) following 12- and 6-h fasts, respectively (26). Blood glucose concentrations were measured at multiple time points (0, 30, 60, 90, and 120 min) using a blood glucose meter (GA-3, Sinocare, Changsha, China) to assess the pharmacodynamics of the administered substances. The area under the blood glucose concentration curve (AUC) at the specified time intervals was used to assess each cohort’s glycemic tolerance and insulin sensitivity.

### 2.4 Biochemical indicators analysis

#### 2.4.1 Serum biochemical analysis

Serum biochemical markers were quantitatively analyzed by employing a colorimetric assay and an automatic biochemistry instrument, following the manufacturer’s instructions (Gracebio, Suzhou, China). The biochemical markers included aspartate aminotransferase (AST), alanine aminotransferase (ALT), high-density lipoprotein (HDL), low-density lipoprotein (LDL), total cholesterol (TC), and triglyceride (TG) levels. ELISA kits were utilized for the determination of LPS, IL-1β, IL-6, TNF-*α*, and Leptin levels.

#### 2.4.2 Liver biochemical assays

Liver samples (500 mg) were homogenized in phosphate-buffered saline (PBS) using a tissue homogenizer and subsequently subjected to centrifugation at 3,000 × g for 15 min at 4°C. The resulting supernatants were then analyzed for concentrations of TG, TC, MDA, SOD, and GSH-Px using commercially available colorimetric assay kits (Gerace Biotechnology Co., Ltd., Suzhou, China).

### 2.5 Histological staining

The liver and ileum tissue specimens were fixed with formalin and then covered with paraffin. The application of hematoxylin and eosin (H&E) staining was performed immediately on the tissue samples. Subsequent examination of the slides was facilitated by means of a Nikon E100 photomicrography imaging device, which enabled the recording of images and the formulation of observations (27). The tissue extracted from the fixed liver was then subjected to desiccation, a process that was deemed essential for the subsequent oil-red O staining experiment. Following this, the specimen was cut on a cryostat after being immersed in an optimum cutting temperature (OCT) embedding agent. The stained specimens were then observed under a 200 × microscope. The histopathological analysis was conducted by investigators who had not been previously informed of the experimental design.

### 2.6 Immunohistochemistry (IHC)

The expression levels of claudin 1, occludin, and occludens zone 1 (ZO-1) proteins in the ileum were evaluated using IHC (immunohistochemistry) as outlined in the extant literature. HRP-conjugated goat anti-rabbit IgG was added after tissue sections were incubated with primary antibodies at a dilution of 1:100. DAB (diaminobenzidine) staining revealed discernible signals, whilst haematoxylin staining delineated the nucleus. The integrated optical density of IHC was measured with ImagePro Plus.

### 2.7 Analysis of the expression of relevant genes and proteins

#### 2.7.1 Quantitative reverse transcription PCR (qRT-PCR)

Total RNA extraction was conducted using the TransZol Up and RNA Extraction Kit. Subsequently, cDNA was generated via the PrimeScript RT Kit, utilising 1 μg of RNA as a template. qPCR amplification reactions were performed using a Roche Light Cycler 480 real-time fluorescent quantitative PCR instrument and Top Green qPCR SuperMix. It is important to note that the relevant reagent system was procured from Beijing All Style Gold Biotechnology Co. in China. The reference gene employed was Actin-*β*, and the quantification of mRNA levels was performed using a 2-ΔΔCT method. The primer sequences are provided in Table 2.

**Table 2:** Primary sequence of the gene.

Target	Sequence
*Accα*	Forward: TCATCCAAACAGAGGGAACA
	Reverse: GTTGTCCAACAGAACATCGC
*Cpt-1*	Forward: CCGAATGTCAAGCCAGACGA
	Reverse: GAGCAGCACCTTCAGCGAGTA
*Fas*	Forward: ACCTCAGCAGCACATCTCAC
	Reverse: CGTCCCTGTACACGTTCATC
*Lxrα*	Forward: TTGCTCTGCTCATAGCCATC
	ReverSe: ATGGAGACATAGGCATGCAG
*Pparα*	Forward: TGAAAGATTCGGAAACTGC
	Reverse: TTCCTGCGAGTATGACCC
*Srebp-1c*	Forward: CGCTACCGTTCCTCTATCAATGAC
	Reverse: AGTTTCTGGTTGCTGTGCTGTAAG
*SHP*	Forward: GGCACTATCCTCTTCAACCCA
	Reverse: TCCAGGACTTCACACAATGCC
*FXR*	Forward: CCACGACCAAGCTATGCAG
	Reverse: TCTCTGTTTGCTGTATGAGTCCA
*NTCP*	Forward: AAAATCAAGCCTCCAAAGGAC
	Reverse: TTGTGGGTACCTTTTTCCAGA
*BSEP*	Forward: CGGTGGCTGAGAGATCAAAT
	Reverse: TGCGATAGTGGTGGAGAACA
β-actin	Forward: AATCCTGCGGCATCCACGAAAC
	Reverse: GTGTTGGCGTAGAGGTCCTTGC
16 sr RNA V3-V4	341F (5’-CCTACGGGNGGCWGCAG-3′)
	805R(5’-GACTACHVGGGTATCTAATCC-3′)

#### 2.7.2 Western blotting analysis

Liver tissues were homogenized in RIPA lysis buffer (Servicebio, Wuhan, China) containing 1 mM phenylmethanesulfonyl fluoride (PMSF). The protein concentrations were then measured using a bicinchoninic acid (BCA) assay kit (Beyotime Biotechnology, Shanghai, China). Aliquots of protein (30–50 μg) were resolved on 8–12% SDS-polyacrylamide gels (Servicebio) and transferred to polyvinylidene fluoride (PVDF) membranes (0.45 μm pore size, Servicebio). The membranes were then blocked with 5% (w/v) non-fat milk in Tris-buffered saline with Tween-20 (TBST) for one hour at room temperature, followed by overnight incubation at 4°C with primary antibodies diluted as follows: p-AMPK (1:1,000), LXRα (1:3,000), SREBP-1c (1:1,000), FAS (1:1,000; Abcam, Cambridge, UK), ACC (1:1,000; Proteintech, Wuhan, China), CPT-1 (1:1,000), PPARα (1:3,000), CYP2E1 (1:1,000), and *β*-actin (1:3,000). Following three TBST washes, the membranes were incubated with horseradish peroxidase (HRP)-conjugated goat anti-rabbit or anti-mouse IgG secondary antibodies (1:3,000, Servicebio) for one hour at room temperature. Protein bands were then visualized using an enhanced chemiluminescence (ECL) reagent (Servicebio) and analyzed with ImageJ software (National Institutes of Health, Bethesda, MD, USA). To ensure the reliability of the results, *β*-actin was used as a loading control. It is noteworthy that each Western blot experiment was performed in triplicate (three independent technical replicates). This was undertaken to ensure the robustness of the data and to provide an adequate margin of safety against the possibility of experimental error. The samples used for this experimentation were obtained from a total of three rats per experimental group and were selected at random.

### 2.8 Gut microbiota analysis

Gut microbiota analysis was performed by collecting fresh faecal samples from the final three days of the intervention. The subsequent extraction of DNA was conducted utilising the NucleoSpin Soil DNA Kit (Macherey-Nagel, Düren, Germany). Primers 341F (5′- CCTACGGGGNGGCWGCAG-3′) and 805R (5′-GACTACHVGGGGTATCTAATCC-3′) were utilized on a Mastercycler Pro Thermal Cycler (Thermo Fisher Scientific, Waltham, MA, USA) for the purpose of PCR amplification of the V3-V4 hypervariable region of the bacterial 16S rRNA gene. The PCR conditions comprised an initial denaturation step for 5 min at 95°C, followed by 35 cycles of 30 s at 95°C, 30 s at 50°C, and 40 s at 72°C, and a final extension step for 7 min at 72°C. The PCR protocol comprised the initial denaturation step at 95°C for 5 min, followed by 35 cycles of 30 s at 95°C, 30 s at 50°C, and 40 s at 72°C. The protocol was concluded with a final extension step at 72°C for 7 min. Amplicons were then purified using the QIAquick PCR Purification Kit (Qiagen, Hilden, Germany) and quantified using a Qubit 4.0 fluorometer (Thermo Fisher Scientific). Equimolar pooled amplicons (2 nM) were then subjected to sequencing on the Illumina MiSeq platform (2 × 300 bp paired-end) from Beijing Biomarker Biotechnology Co. The resulting raw sequences were then pair-end merged using FLASH v1.2.11 and subjected to a quality filter using Trimmomatic v0.33 (sliding window: 4 bp; average quality > 20). Microbiota data were analyzed using Amplicon Sequence Variant (ASV). Classification assignments were made according to the SILVA 138.1database. Alpha diversity (Shannon and Simpson indices) and beta diversity (PCoA, NMDS based on unweighted UniFrac distances) were calculated using QIIME2 v2022.8. Linear discriminant analysis effect size (LEfSe) was utilized to identify differentially abundant taxa (LDA score > 3) from phylum to genus. Group comparisons employed the Kruskal-Wallis test (*p* < 0.05).

### 2.9 Statistical analysis

All data were analyzed using GraphPad Prism 10 (San Diego, CA, USA) and are expressed as mean ± standard deviation (SD) unless otherwise specified. For the gut microbiome 16S rRNA sequencing data, principal component analysis (PCA) was performed and visualised using the R language platform (vegan package v 2.6–4). The assessment of between-group differences was conducted via one-way analysis of variance (ANOVA), followed by Tukey’s *post hoc* test for multi-group comparisons. Statistical significance was defined at *p* < 0.05.

## 3 Results

### 3.1 HEH and MEH are more effective in improving HFD-induced obesity

**Figure 1:**
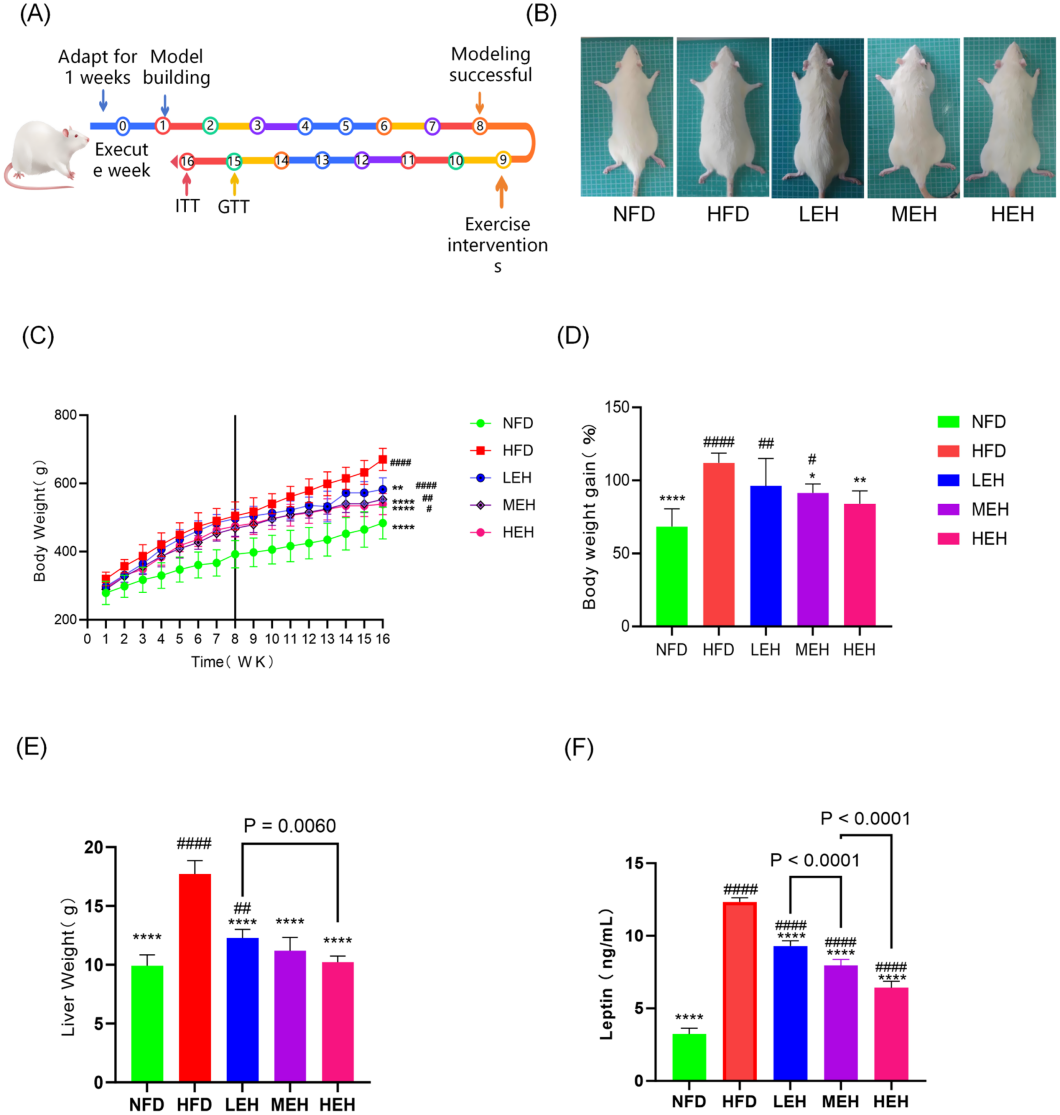
Effects of exercise on body weight and body fat content of HFD rats: **(A)** Flowchart of the experiment; **(B)** The fundamental requirements for maintaining a colony of rats; **(C)** weight gain curve, **(D)** body weight growth of rats, **(E)** hepatic wet weight, **(F)** Blood Leptin levels, each value is expressed as mean ± SD (*n* = 6), *p* < 0.05(*), *p* < 0.01(**), *p* < 0.001(***), and *p* < 0.0001(****) in relation to the HFD group; *p* < 0.05(#), *p* < 0.01(##), *p* < 0.001(###), and *p* < 0.0001(####) in relation to the NFD group.

The weight loss effect could be observed with different loads of aerobic exercise (Figure 1A). After 8 weeks of exercise intervention, the mean body weight of rats fed high-fat chow was significantly increased by about 112.1% (353.8 g) compared to the first week, whereas the mean body weight of rats on normal diet was only increased by about 68.4% (195.5 g), and the mean body weight of rats that underwent LEH was increased by 96.2% (284.4 g), the mean body weight of rats in the HEH group was increased by 91.4% (264.1 g), and rats in the HEH group gained an average of 84.1% (246.3 g). Comparing the HFD groups, HEH was more able to attenuate HFD-induced weight gain relative to LEH and MEH (Figures 1A,B). In addition, all three exercise groups significantly suppressed HFD-induced epididymal fat volume (*p* < 0.01) (Figures 1C,D). HEH and MEH reduced epididymal fat weight more significantly than LEH (*p* < 0.05).

### 3.2 HEH and MEH improved hepatic lipid degeneration better than LEH

**Figure 2:**
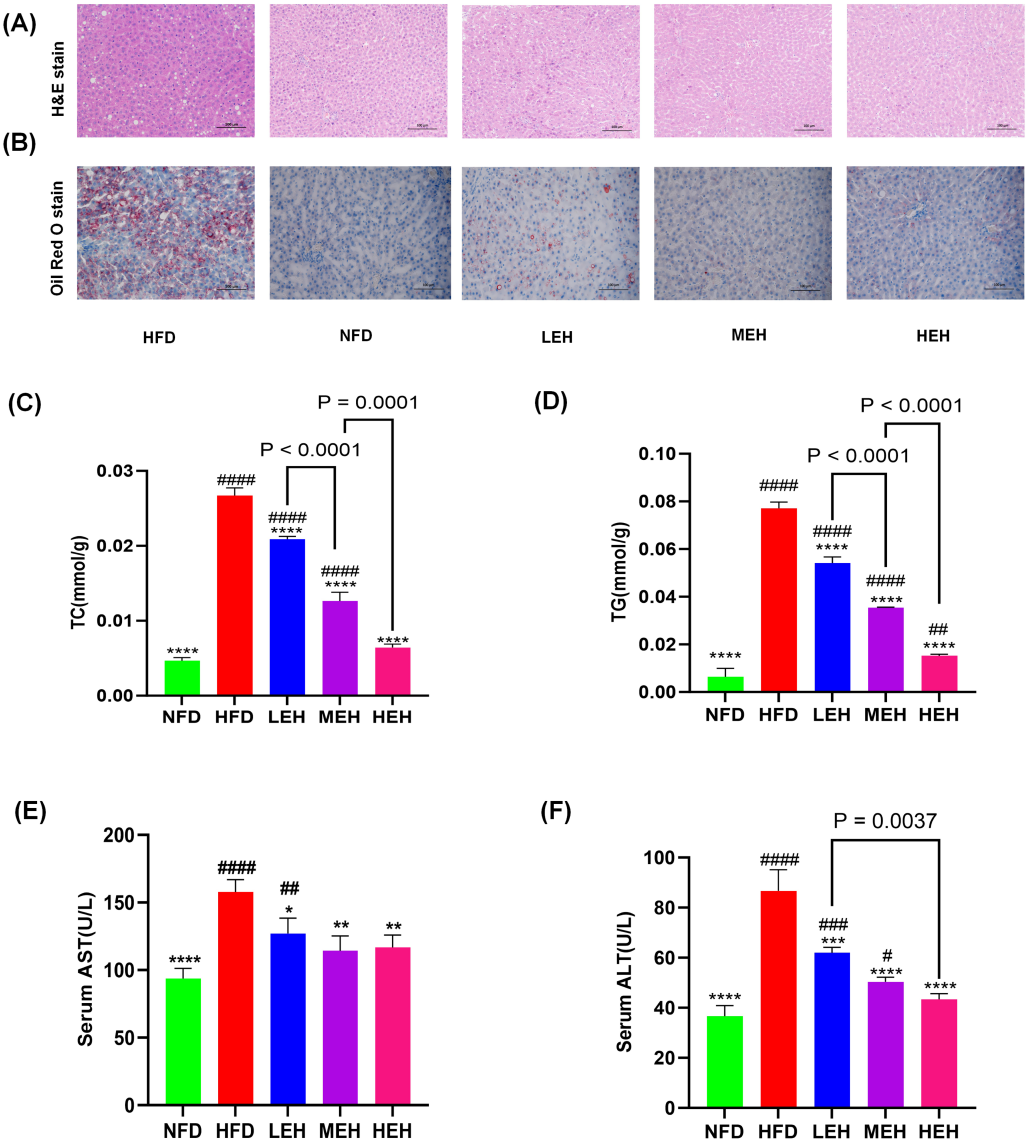
Effects of exercise on hepatic steatosis and lipid metabolism in HFD rats: **(A,B)** H&E and oil red O staining of liver tissue sections, **(C,D)** A comparison of the concentrations of liver TG and Total TC in various groups, and **(E,F)** concentrations of enzymes related to hepatic function. Each value is expressed as mean ± SD (*n* = 6), *p* < 0.05(*), *p* < 0.01(**), *p* < 0.001(***), and *p* < 0.0001(****) in relation to the HFD group; *p* < 0.05(#), *p* < 0.01(##), *p* < 0.001(###), and *p* < 0.0001(####) in relation to the NFD group.

After exercise intervention, HFD-induced accumulation of lipid vesicles and lipid droplets was significantly reduced (Figures 2A,B). Compared with the NFD group, hepatic lipid content (TG, TC) and liver function indexes (ALT, AST) were significantly elevated in the HFD group. After performing three groups of exercise training with different loads, the hepatic lipid levels gradually returned to normal levels, and ALT and AST levels were significantly reduced (Figures 2C–F). In addition, MEH had a more significant improvement effect on TC and TG indexes compared with LEH (*p* < 0.001), and HEH had a more significant improvement effect on TC and TG indexes compared with MEH (*p* < 0.001).

### 3.3 HEH is better at regulating lipid metabolism

**Figure 3:**
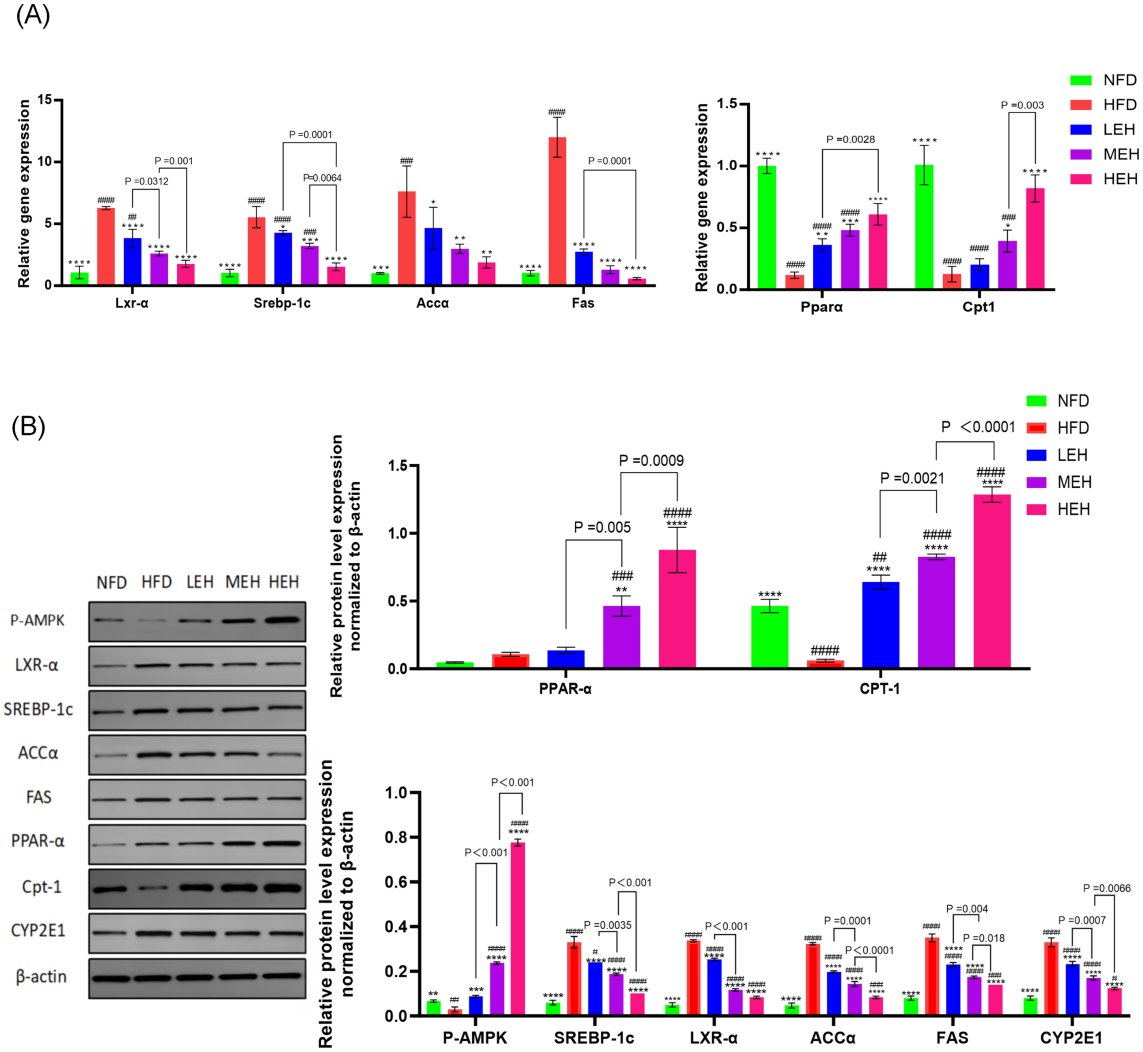
**(A,B)** Effects of exercise on the expression of factors related to hepatic lipid synthesis and lipid oxidation in HFD rats. Each value is expressed as mean ± SD, *p* < 0.05(*), *p* < 0.01(**), *p* < 0.001(***), and *p* < 0.0001(****) in relation to the HFD group; *p* < 0.05(#), *p* < 0.01(##), *p* < 0.001(###), and *p* < 0.0001(####) in relation to the NFD group.

The results showed that the liver wet weight and Leptin level of rats in HFD group were significantly higher than that of NFD (*p* < 0.0001). MEH was better at improving Leptin level than LEH, while HEH was more effective at improving Leptin level compared with MEH (Figure 1F). The present study further measured the effects of three sets of aerobic exercise with different loads on several representative lipid metabolism genes and proteins. All three sets of exercise suppressed Lxrα and its downstream lipid synthesis-related genes (*Srebp-1c*, *Fas* and *Accα*), while increasing the expression of lipid degradation-related genes (*PPARα* and *CPT-1*). In addition, exercise activated the phosphorylation of AMPK and inhibited the overexpression of lipid peroxidation regulatory protein (Cytochrome P450 2E1, CYP2E1) (Figure 3). In addition, HEH was better at regulating lipid metabolism compared to the three aerobic exercise groups.

### 3.4 Three groups of exercise improved HFD-induced glucose tolerance and insulin resistance

**Figure 4:**
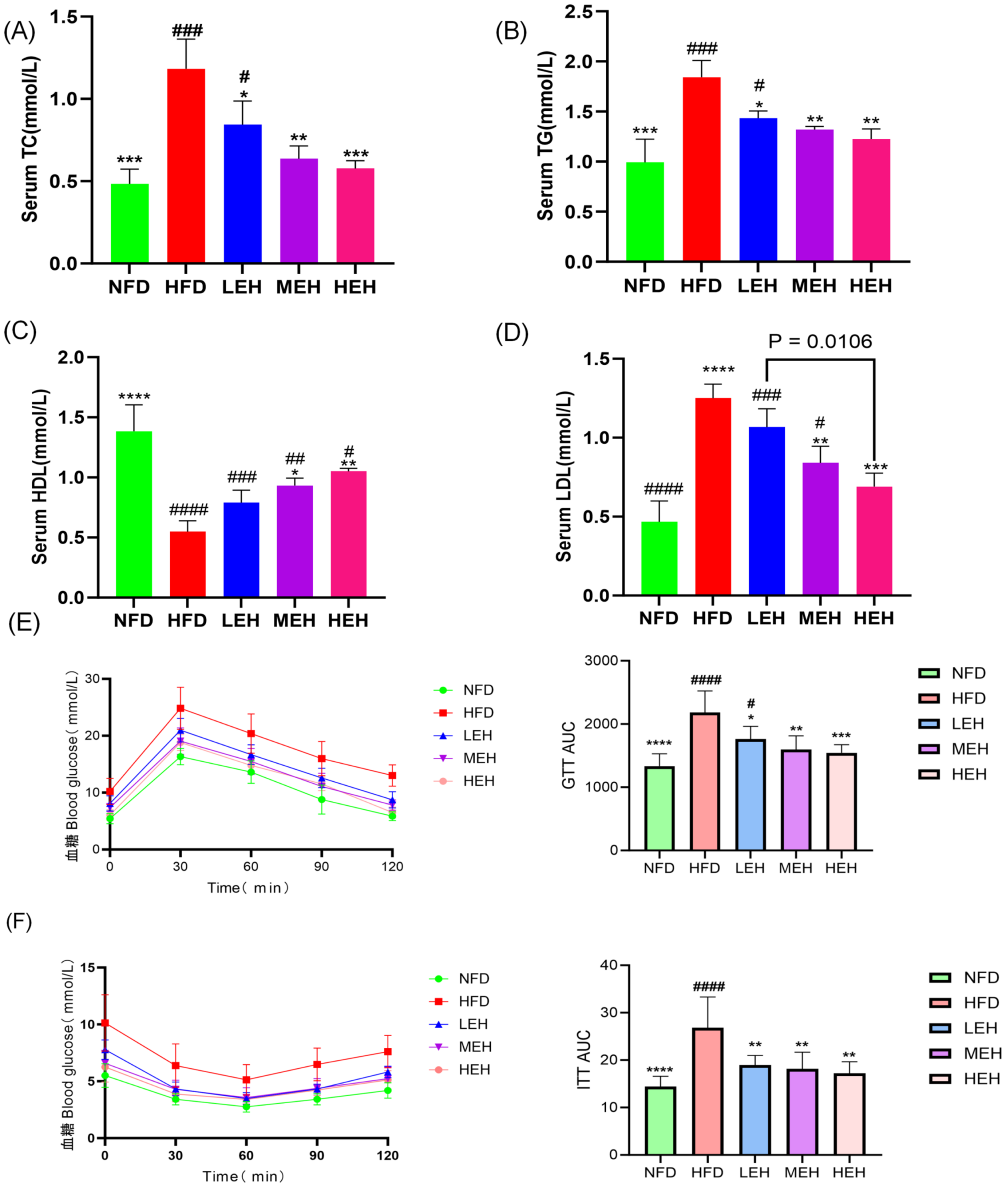
Effect of exercise on insulin resistance in HFD rats: **(A)** Triglycerides, **(B)** Total cholesterol, **(C)** HDL-C, **(D)** LDL-C, **(E)** GTT, GTT AUC, and **(F)** ITT, ITT AUC. Each value is expressed as mean ± SD (*n* = 6). *p* < 0.05(*), *p* < 0.01(**), *p* < 0.001(***), and *p* < 0.0001(****) in relation to the HFD group; *p* < 0.05(#), *p* < 0.01(##), *p* < 0.001(###), and *p* < 0.0001(####) in relation to the NFD group.

HFD significantly increased serum TC, TG, and LDL levels, whereas serum HDL levels were significantly decreased, and exercise significantly improved dyslipidemia (Figures 4A–D). The areas under the GTT and ITT curves confirmed that HFD-induced abnormalities of glucose tolerance and insulin resistance were significantly improved by the three-group aerobic exercise intervention.

### 3.5 Exercise relieves oxidative stress in the liver

**Figure 5:**
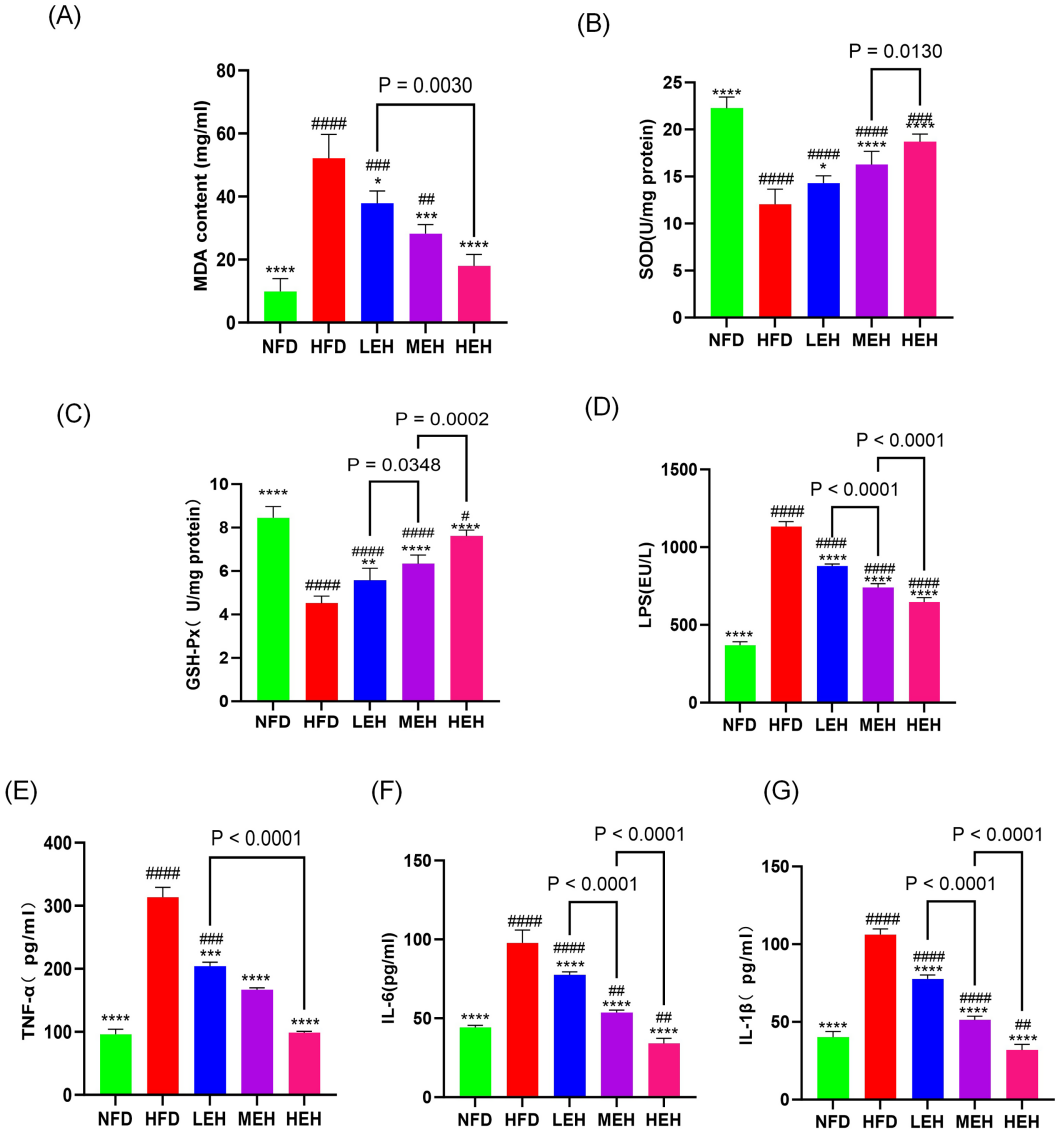
Exercise administration relieves oxidative stress in the liver and systemic inflammation: **(A–C)** MDA content and antioxidant enzyme activity in the liver, **(D)** LPS levels, **(E–G)** serum concentrations of TNF-α, IL-1*β*, and IL-6. Each value is expressed as mean ± SD (*n* = 6), *p* < 0.05(*), *p* < 0.01(**), *p* < 0.001(***), and *p* < 0.0001 (****) in relation to the HFD group; *p* < 0.05(#), *p* < 0.01(##), *p* < 0.001(###), and *p* < 0.0001(####) in relation to the NFD group.

In liver biochemical analysis, lipid peroxides malondialdehyde content, superoxide dismutase content, and glutathione peroxidase activity decreased with exercise intervention, and HEH was the most effective in modulating hepatic oxidative stress among the three groups of exercise (Figures 5A–C).

### 3.6 MEH and HEH were more effective in reducing endotoxemia and systemic inflammation by enhancing intestinal barrier function than LEH

**Figure 6:**
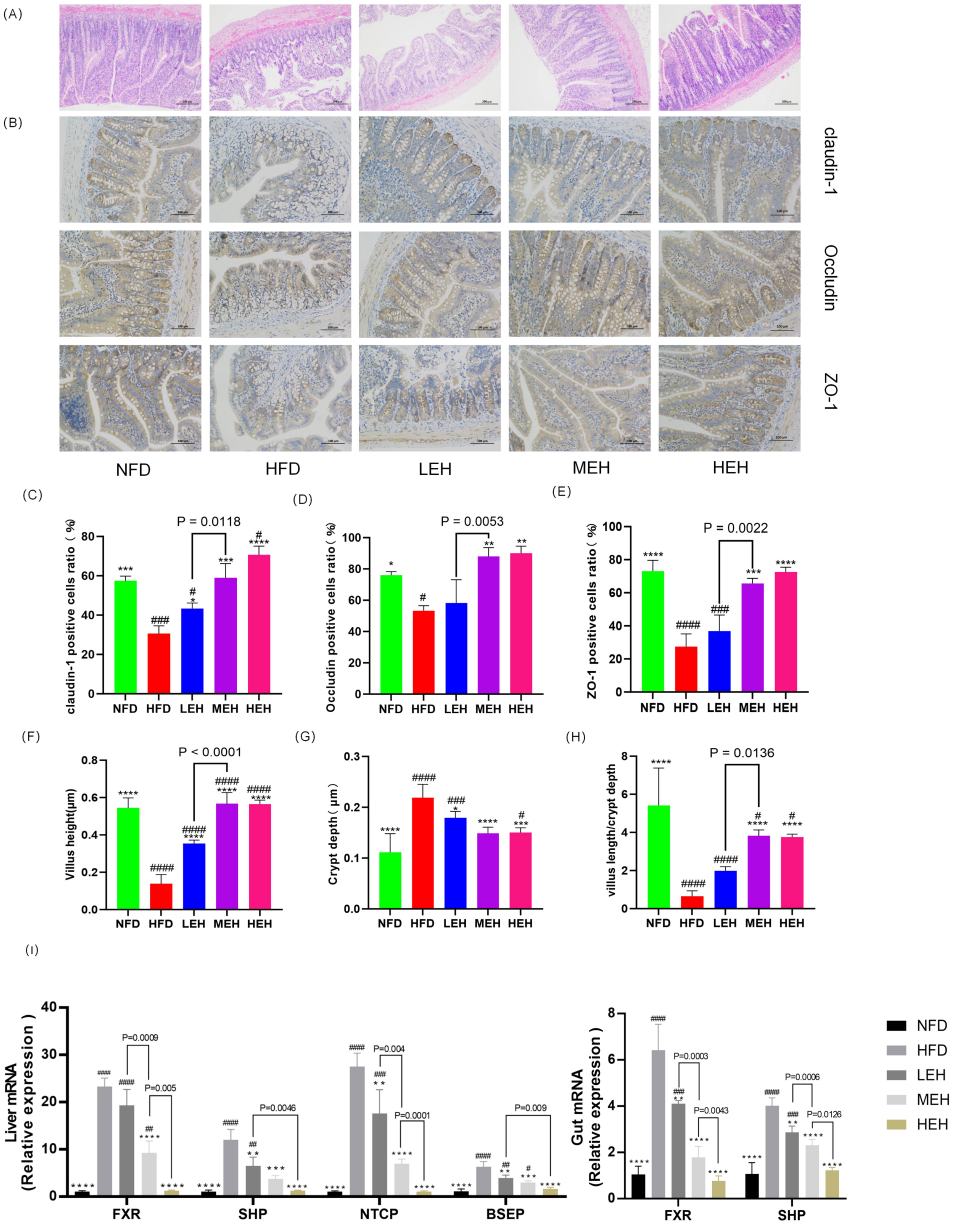
Exercise has been shown to be effective in attenuating endotoxemia by enhancing intestinal barrier function and improving enterohepatic circulation through the bile acid pathway. **(A)** H&E staining of representative ileal sections, **(B)** IHC of ileal tight junction proteins, **(C–E)** immunohistochemical quantitative values of ileal Claudin-1, Occludin, and ZO-1 proteins, respectively, and **(F–H)** showing villus height, crypt depth, and ratio, **(I)** mRNA levels of genes related to BA metabolism. Each value is expressed as mean ± SD (*n* = 6), *p* < 0.05(*), *p* < 0.01(**), *p* < 0.001(***), and *p* < 0.0001(****) in relation to the HFD group; *p* < 0.05(#), *p* < 0.01(##), *p* < 0.001(###), and *p* < 0.0001(####) in relation to the NFD group.

By blood biochemical analysis, it was found that exercise significantly reduced the serum levels of LPS and pro-inflammatory factors (Figures 5D–G), indicating that exercise could effectively alleviate HFD-stimulated endotoxemia and systemic inflammation and that HEH had the best effect among the three exercise groups. In addition, exercise improved the intestinal epithelial structure of the ileum (Figures 6A, F–H), effectively attenuating HFD-induced loss of intestinal tight junction proteins (Figures 6B–E). Importantly, compared with the LEH group, the MEH group had a significant effect on the restoration of ileal villus length and crypt depth and a better effect on the improvement of intestinal epithelial structure.

### 3.7 Exercise ameliorates HFD-induced obesity and hepatic steatosis by modulating BA metabolism and enterohepatic circulation

HFD-induced rats deregulated hepatic BA uptake and significantly upregulated the expression of Na + −taurocholate cotransporting polypeptide (*NTCP*) and bile salt export pump (*BSEP*). These effects were associated with enhanced hepatic expression of the nuclear Farnesoid X Receptor (*FXR*), leading to a significant induction of the small heterodimer partner (*SHP*) in HFD-induced impairment of the enterohepatic BA cycle by a mechanism involving microbiota composition and dysfunction. Among them, *FXR* appeared to be upregulated, leading to increased *SHP* expression. The exercise intervention group restored HFD-induced hepatic and intestinal *FXR* expression levels to those observed in sedentary control rats, which eliminated hepatic and intestinal SHP gene expression and similarly counteracted HFD-mediated NTCP and *BSEP* hepatic overexpression (Figure 5I). Notably, MEH and HEH were superior to LEH for the regulation of genes related to bile acid metabolism.

### 3.8 Exercise training alters gut microbiota composition in HFD rats

**Figure 7:**
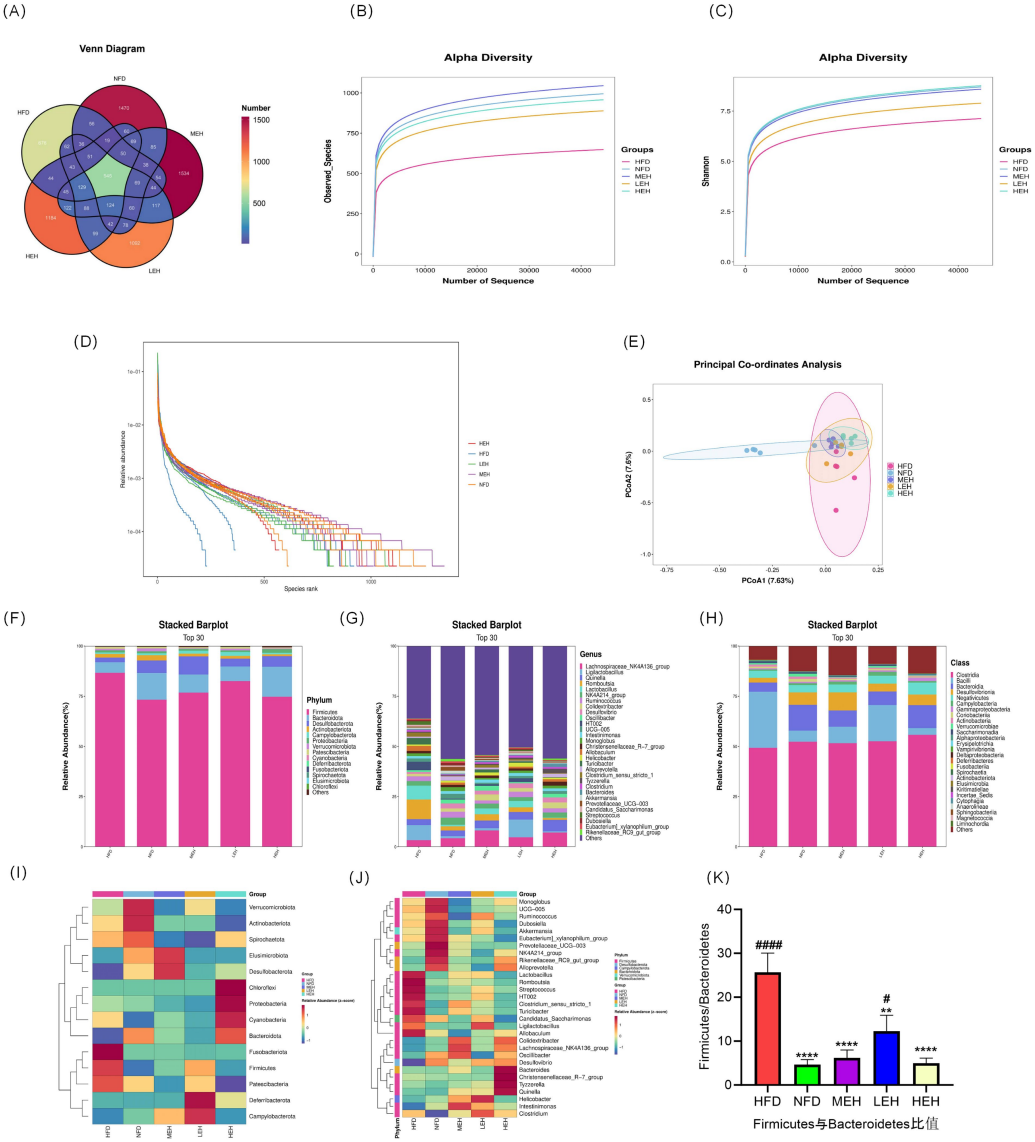
Effects of exercise on the diversity, abundance and structure of gut flora in obese rats: **(A–D)** Venn diagrams, observed species curves for each group, Shannon curves for each group and OTU ranking curves; **(E)** Jaccard-based PCoA score plots; **(F)** Relative abundance of intestinal microbial communities at phylum and genus levels; **(F, G)** Relative abundance of intestinal microbial communities at phylum levels; **(H)** Relative abundance of intestinal microbial communities at phylum levels; **(I, J)** Heatmaps showing clustering (mean) of the relative abundance of all groups of microbial communities at phylum and genus levels; **(K)** Thick walled bacteria phylum/anabolic bacillus ratio abundance; **(I, J)** heatmap showing clustering of relative abundance of microbial communities at the phylum and genus level for all groups (mean); **(K)** *Firmicutes/Bacteroidetes* ratio. *p* < 0.05 (*), *p* < 0.01 (**), *p* < 0.001 (***), *p* < 0.0001 (****) relative to the HFD group. *p* < 0.05 (#), *p* < 0.01 (##), *p* < 0.001 (###), *p* < 0.0001 (####) relative to the NFD group.

A total of 545 common OTUs were found in the four groups, 676 specific OTUs in the HFD group, 1,470 specific OTUs in the NFD group, 1,092 specific OTUs in the LEH group, 1,534 specific OTUs in the MEH group, and 1,184 specific OTUs in the MEH group. The HFD diet significantly decreased the abundance of gut flora, and the results of the PCoA of the OTU levels showed a significant separation effect between the normal and the model groups, while the LEH, MEH, and HEH groups were closer to the NFD group. Exercise significantly induced a change in the gut microbiota composition in HFD rats. Groups were separated significantly. LEH, MEH, and HEH groups were closer to NFD. Exercise significantly induced the abundance and diversity of gut microbiota in HFD rats, with microbiota composition tending to favor the NFD group (Figures 7A–E). According to the histograms as well as heatmaps, at the phylum level, Fusobacteriota and *Firmicutes* abundance as well as *Firmicutes*/*Bacteroidetes* ratio were significantly higher in the HFD group compared to the NFD group (Figures 7E,J), and after the exercise intervention, the abundance of thick-walled *Firmicutes* and the *Firmicutes*/*Bacteroidetes* ratio were significantly lower and the MEH and HEH groups were more convergent to the NFD level (Figure 7K); the MEH and HEH groups were more convergent to the NFD level (Figure 7K). phylum (*Bacteroidetes*) ratio as well as *Fusobacteriota* abundance were significantly lower and more convergent to the NFD level in the MEH and HEH groups (Figure 7K); at the phylum level, the expression of *Clostridia* and *Bacteroidia* was significantly lower in the HFD group compared to the NFD group, and the same three groups of aerobic exercise reversed the above situation (Figure 6J); at the family level, *Oscillospiraceae* had lower expression in the HFD group compared to the NFD group; at the genus level, the HFD group showed that *Ruminococcaceae_NK4A214_group*, *Lachnospiraceae_NK4A136_group*, *Colidextribacter*, *Oscillibacter*, *Prevotellaceae_UCG-003*, *UCG-005*, *Akkermansia* decreased in abundance, *Romboutsia*, *Clostridium_sensu_stricto_1*, *Turicibacter*, *Allobaculum* increased in abundance, and the HFD-induced abnormal pattern of abundance of the above phyla was somewhat undone by the exercise intervention (Figures 7B,D), and it is noteworthy that the improvement effect of exercise on most of the phyla was best in the MEH, HEH group.

### 3.9 Correlation of gut flora composition with obesity-associated MASLD profiles

**Figure 8:**
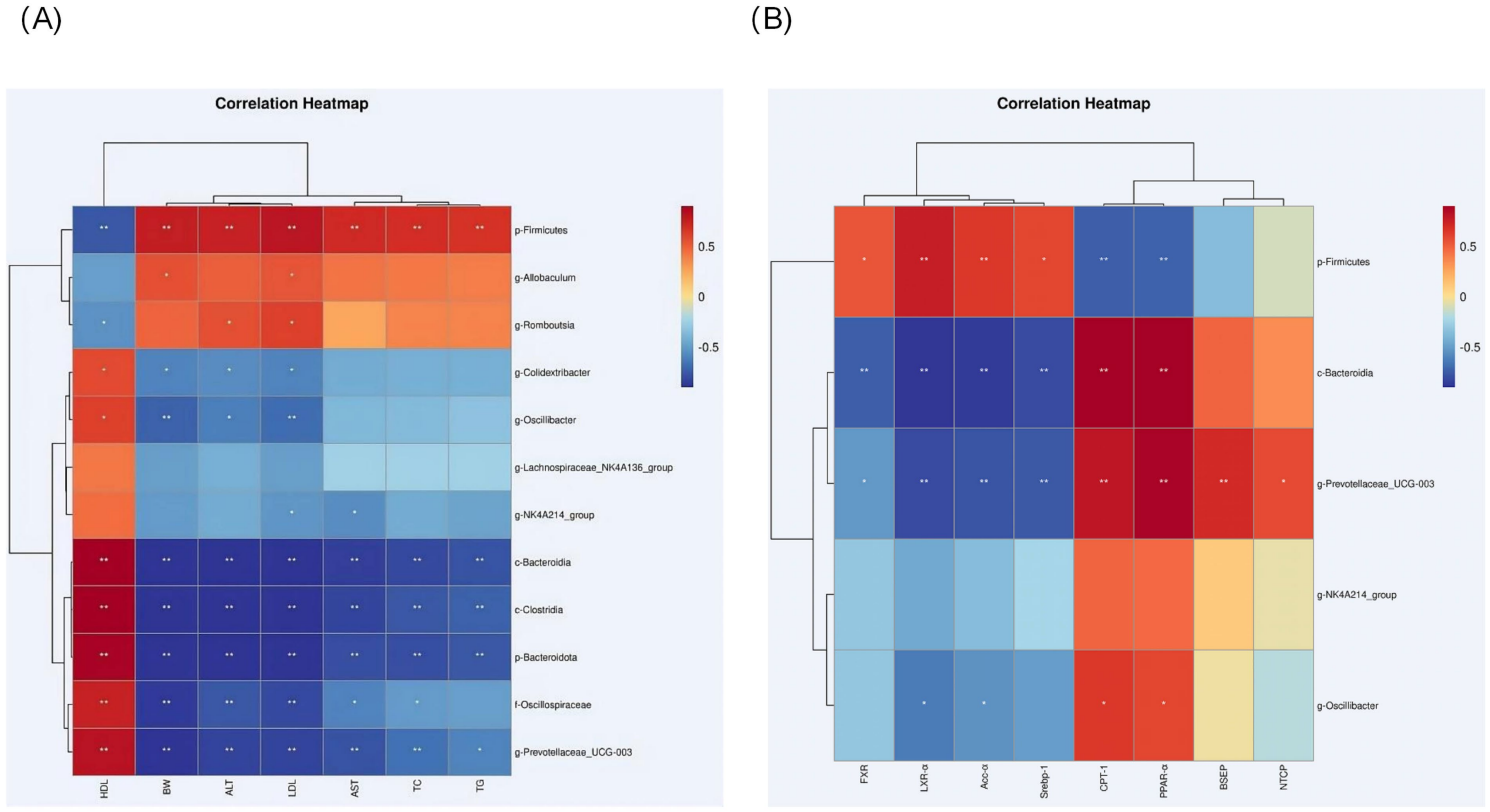
**(A)** Heatmap showing correlations between selected genera of differential abundance and MASLD-related metrics in each group; and correlations were analyzed using bilateral Spearman’s correlation (*, ** indicate *p* < 0.05 and 0.01, respectively). **(B)** Heatmap showing correlations between selected genera of differential abundance and lipid metabolism-related metrics and bile acid pathway-related metrics in each group; and correlations were analyzed using bilateral Spearman’s correlation (*, ** indicate *p* < 0.05 and 0.01, respectively).

In the obesity and MASLD model, the relative abundance of *Bacteroidia*, *Clostridia*, *Prevotellaceae_UCG-003* and *Oscillibacter* was negatively correlated with weight gain (*p* < 0.05), ALT (*p* < 0.05), AST (*p* < 0.05) and LDL (*p* < 0.05) plasma levels were negatively correlated with and positively correlated with HDL levels (*p* < 0.05). *Oscillibacter* and *Colidextribacter* were also negatively correlated with LDL levels (*p* < 0.05), body weight (*p* < 0.05), and ALT (*p* < 0.05) levels, and positively correlated with HDL; *Firmicutes*, *Rumboutsia* abundance was negatively correlated with HDL levels, where *Firmicutes* was positively correlated with body weight gain (*p* < 0.05), TC (*p* < 0.05), TG (*p* < 0.05), ALT (*p* < 0.05) and AST (*p* < 0.05) plasma levels, *Allobaculum* was positively correlated with body weight (*p* < 0.01), LDL (*p* < 0.05) correlation, and *Romboutsia* was positively correlated with ALT (*p <* 0.01) and LDL (*p* < 0.05) plasma levels (Figure 8A).

The abundance of *Bacteroidia*, *Prevotellaceae_UCG-003* was positively correlated with lipolytic genes CPT-1, PPAR-*α* (*p* < 0.01), where UCG-003 was positively correlated with bile acid pathway related genes NTCP, BSEP (*p* < 0.01), and negatively correlated with adipogenic genes SREBP-1, ACC-α, LXR-α (*p* < 0.01). ACC-α, LXR-α (*p* < 0.01), both of which were negatively correlated with bile acid pathway-related genes FXR (*p* < 0.01); the abundance of firmicutes was positively correlated with the levels of LXR-α, SREBP-1, ACC-α and FXR (*p* < 0.05), and negatively correlated with the levels of CPT-1 (*p* < 0.05), PPAR-α (*p* < 0.01). *Oscillibacter* abundance was negatively correlated with the levels of LXR-α, ACC-α (*p* < 0.05), and positively correlated with the levels of CPT-1 and PPAR-α (*p* < 0.05) (Figure 8B).

## 4 Discussion

In recent years, the role of intestinal-hepatic axis dysfunction in the pathogenesis of MASLD has attracted much attention, and its pathological process involves several key links, such as abnormal oxidative stress and disruption of the dynamic balance of lipid metabolism (28, 29). In this study, we systematically analyzed the intervention effects of aerobic exercise of different intensities and found that exercise training can produce multi-target protective effects on MASLD by regulating lipid metabolic pathways and repairing intestinal-hepatic axis homeostasis. In the present study, we found that long-term HFD resulted in significant weight gain (Figures 1C,D), insulin resistance and impaired gut function (30). Further analyses showed that all three training modes slowed down the MASLD process by reducing oxidative stress (Figures 5A–C), modulating serum lipid composition (decreasing TG and LDL-C and increasing HDL-C) (Figures 4A–D) and improving insulin sensitivity and glucose tolerance (16, 26, 27) (Figures 4E,F). And the three exercises decreased serum leptin levels (Figure 1E) (31). Specifically, high- and medium-intensity exercise (HEH, MEH) showed significantly better effects than low-intensity exercise (LEH) in reducing obesity-related weight gain (Figures 1C,D), improving serum aminotransferase levels (Figures 2E,F), and regulating the expression of key genes for bile acid metabolism (Figure 6I), suggesting that the dose effect of exercise is clinically important in metabolic regulation. From the molecular mechanism level, exercise intervention can synergistically inhibit the lipid synthesis axis of SREBP1c/FAS/ACCα/LXRα and activate the fatty acid oxidation pathway of PPARα/CPT-1 to achieve dual regulation (32), in which the AMPK signaling pathway, as a core hub of energy metabolism, accelerates the inactivation process of the ACC proteins by phosphorylating them, and then breaks down the lipid synthesis and catabolism. Which in turn breaks the imbalance between lipid synthesis and catabolism. Notably, the exercise-induced phosphorylation cascade of cyclophosphoadenosine effector binding protein (CREB) significantly enhanced the mitochondrial biosynthesis capacity of hepatocytes, which provided a new molecular basis for elucidating the amelioration of hepatic lipid oxidative stress by exercise (33).

As the anatomical basis for the functional maintenance of the gut-liver axis, the damage and repair mechanisms of the intestinal barrier integrity have an important role in the evolution of the MASLD disease process (9). In the present experiment, we observed that high-fat diet feeding significantly down-regulated the expression levels of the tight junction proteins claudin1, occludin, and ZO-1, and this intestinal mucosal structural damage could be effectively reversed by exercise intervention (Figures 6A–H). The underlying mechanisms may involve the reduction of circulating endotoxin levels and inhibition of CYP2E1-mediated lipid peroxidation by exercise training, while restoring the activity of endogenous antioxidant systems such as superoxide dismutase (SOD) and glutathione peroxidase (GSH-Px) (34–37). At the microbial community level, the ecological imbalance of intestinal flora caused by a high-fat diet is manifested by an imbalance in the ratio of Thick-walled phylum/Anthrobacterium and an abnormal increase in the abundance of *Clostridium* (38, 39), whereas regular exercise can reshape the intestinal microenvironment, which is specifically reflected by the proliferative dominance of butyric acid-producing bacterial flora (e.g., *Ruminalcoccaceae*, *Trichoderma*) and the suppression of the abundance of pro-inflammatory genera, such as *Romboutsia* (40, 41). In-depth analysis revealed that exercise-induced amplification of butyric acid-producing bacteria could promote hepatic fatty acid *β*-oxidation process through activation of the AMPK-ACC signaling pathway (42), and at the same time regulate intestinal epithelial lipid uptake metabolism via the PPAR-ANGPTL4 axis (43, 44), and the elucidation of the mechanism of this colony-host metabolism interplay provides a new theoretical perspective for the understanding of the amelioration of lipid disorders by exercise.

Comparative studies of the effects of interventions with different exercise intensities reveal dose-dependent biological features. High-load aerobic exercise (HEH) showed optimal effects in alleviating hepatic lipid deposition, improving leptin resistance, and repairing intestinal tight junction structures, which was closely related to the significant reduction of serum leptin levels and inhibition of TGF-β signaling pathway induced by high-intensity exercise (45–49). Although moderate-intensity exercise (MEH) was slightly inferior to HEH in terms of absolute effect values, its regulation of genes related to bile acid metabolism was still significantly better than that of low-intensity exercise (LEH), which may be related to the improvement of enterohepatic recycling of bile acids through the specific activation of FXR-SHP signaling cascade response by MEH (50–52). Differences at the mechanistic level may stem from differences in the activation of the CREB/cAMP signaling pathway by exercise intensity, with high-intensity exercise enhancing lipid oxidation via more pronounced mitochondrial biosynthesis, whereas moderate-intensity exercise achieves regulation of metabolic homeostasis by optimizing the efficiency of energy metabolism (53). Notably, exercise-induced gut flora remodeling showed a significant intensity gradient, with HEH and MEH promoting the proliferation of metabolically protective genera such as *Oscillospira* more than LEH, and the abundance of this genus was significantly negatively correlated with hepatic adiposity (54, 55), which provides a potential direction for the development of a system to assess the efficacy of exercise based on gut microbial markers.

In this study, we systematically elucidated the multidimensional action network of exercise intervention to improve MASLD at the molecular, cellular, and organ levels and confirmed that exercise training exerts therapeutic effects through the synergistic effects of lipid metabolism reprogramming, intestinal barrier repair, and ecological remodeling of bacterial flora. These findings not only provide theoretical support for the development of precise exercise prescription but also open up new ideas for the development of metabolic disease intervention strategies targeting the gut-liver axis. Follow-up studies are needed to further analyze the dynamic relationship between exercise intensity and the metabolites of intestinal flora and to explore the specific mechanisms of specific strains and their metabolites in the exercise-mediated metabolic improvement.

### 4.1 Strengths and limitations

This study has several strengths, not the least of which is its design based on the enterohepatic axis, which sets it apart from other studies in the field. By comparing different types of exercise in the context of MASLD, it provides a more comprehensive assessment of the bile acid pathway, lipid metabolism, and other relevant metrics. It also explains possible mechanisms. However, it is also important to understand its limitations. For example, it did not assess liver function markers and genes associated with lipid metabolism prior to training, and it lacked baseline data, which could affect the reliability of the results. In addition, it did not examine how lipid-related processes in the body are affected during training. Therefore, we were unable to get a full picture of the effects of the three exercises on these processes. This study also did not perform a lipidomics study to further clarify the effects of exercise on lipogenesis.

## 5 Conclusion

In conclusion, the present study demonstrated that the HEH and MEH groups were better at improving HFD-induced obesity as well as improving glucose tolerance and insulin tolerance, and were more effective at restoring the length of ileocecal villi and crypt depth as well as the abundance of intestinal flora compared with the LEH group, suggesting that the effect of improving the intestinal epithelial structure was better. What’s more, to some extent, the HEH group was superior in improving obesity, liver function, and lipid metabolism, as well as regulating Leptin and LPS levels compared to the MEH group. Therefore, considering the time cost and the efficacy of various loads of exercise, a more scientific and rigorous exercise prescription should be developed for MASLD.

## Data Availability

The datasets presented in this study can be found in online repositories. The names of the repository/repositories and accession number(s) can be found in the article/supplementary material.
